# The effects of a maternal nursing competency reinforcement program on nursing students’ problem-solving ability, emotional intelligence, self-directed learning ability, and maternal nursing performance in Korea: a randomized controlled trial

**DOI:** 10.4069/kjwhn.2021.09.13

**Published:** 2021-09-30

**Authors:** Sun-Hee Kim, Bo Gyeong Lee

**Affiliations:** College of Nursing, Research Institute of Nursing Science, Daegu Catholic University, Daegu, Korea

**Keywords:** Emotional intelligence, Maternal-child nursing, Problem solving, Self-directed learning as topic, Students

## Abstract

**Purpose:**

The purpose of this study was to develop a maternal nursing competency reinforcement program for nursing students and assess the program’s effectiveness in Korea.

**Methods:**

The maternal nursing competency reinforcement program was developed following the ADDIE model. This study employed an explanatory sequential mixed methods design that applied a non-blinded, randomized controlled trial with nursing students (28 experimental, 33 control) followed by open-ended interviews with a subset (n=7). Data were analyzed by both qualitative and quantitative analysis methods.

**Results:**

Repeated measures analysis of variance showed that significant differences according to group and time in maternal nursing performance; assessment of and intervention in postpartum uterine involution and vaginal discharge (F=24.04, *p*<.001), assessment of and intervention in amniotic membrane rupture (F=36.39, *p*<.001), assessment of and intervention in delivery process through vaginal examination (F=32.42, *p*<.001), and nursing care of patients undergoing induced labor (F=48.03, *p*<.001). Group and time improvements were also noted for problem-solving ability (F=9.73, *p*<.001) and emotional intelligence (F=4.32, *p*=.016). There were significant differences between groups in self-directed learning ability (F=13.09 *p*=.001), but not over time. The three themes derived from content analysis include “learning with a colleague by simulation promotes self-reflection and learning,” “improvement in maternal nursing knowledge and performance by learning various countermeasures,” and “learning of emotionally supportive care, but being insufficient.”

**Conclusion:**

The maternal nursing competency reinforcement program can be effectively utilized to improve maternal nursing performance, problem-solving ability, and emotional intelligence for nursing students.

## Introduction

In the current clinical practice education environment, as the awareness of patient rights and the demand for nursing services increase, nursing students only observe patients in the clinical field rather than learning actual nursing skills [1]. In addition, in Korea, there are relatively few cases of pregnancy and childbirth because of the extremely low fertility rate. Also, patients often feel uncomfortable with exposing their sensitive areas and health care providers need to protect patient privacy, so it is particularly difficult for nursing students to practice in delivery rooms [2]. Furthermore, it is difficult for nursing students in practicum to continuously observe and practice delivery-related care because delivery is a long and continuous process [3]. Thus, nursing students often lack sufficient opportunity to perform the necessary health assessments during pregnancy, childbirth, and postpartum stages as well as, interpret the results and apply nursing care needed for the situation. As a result, nursing students may have difficulty in solving nursing-related problems and performing as nurses after graduation [4].

In Korea, simulation is often reinforced in the nursing curriculum to overcome the limitations of clinical practice education and improve the problem-solving abilities of students in various nursing situations [5]. According to previous studies, simulation education had a positive relationship with nursing students’ knowledge [6], self-efficacy [6], clinical performance ability [7], problem-solving ability [5], clinical judgment ability [8], and self-directed learning ability [9]. However, since previous studies on simulation programs for maternal nursing were lacking, more studies are needed to verify simulation training programs for maternity nursing and determine their effectiveness. In particular, as Korea’s fertility rate is very low and clinical practice alone is not sufficient in maternal nursing education, simulation allows students to practice real nursing skills [2]. Moreover, in the era of the 4th Industrial Revolution, nurses are required to have problem-solving skills to solve complex problems as well as self-direction to cope with the ever-changing environment [10]. Since emotional intelligence is related to clinical performance [11] and problem-solving ability [12], nursing students need to improve their emotional intelligence.

In order to provide an education that is tailored to each student population, it is useful to reflect on the level and needs of the population concerning the educational subject at hand. If an educational program does not reflect the students’ performance level, its efficacy may be reduced for learners who have already reached high achievement levels. Conversely, it can cause frustration for low-achieving learners.

This study aimed to develop and evaluate a maternal nursing competency reinforcement program based on the core principles of maternal nursing that considers the students’ needs and levels.

## Methods

Ethics statement: This study was approved by the Institutional Review Board of Daegu Catholic University (CUIRB-2018-0040). Written consent was obtained from students who indicated voluntary participation. An explanation was provided to students that refusal to participate in the study did not adversely affect grades.

### Program development phase

The ADDIE (Analysis, Design, Development, Implementation, and Evaluation) model was used to select, apply, and evaluate the elements of core nursing care related to childbirth [13] (Supplementary Fig. 1).

#### Analysis phase

For analysis of learning needs and job tasks for maternal nursing competency, the researchers selected 15 maternal nursing skills through a literature review and developed a structured questionnaire. The questionnaire was used to investigate the importance of maternal care skills and the amount of time required to perform those skills, both in the present and in the future. Data collection took place from September 14 to 24, 2018. Structured questionnaires were collected from experts (n=8) and third year nursing students (n=83), followed by focus group interviews with students (n=19).

#### Design phase

Based on the analysis phase, performance goals were determined as of core nursing competency related to childbirth, self-directed learning, emotional intelligence utilization, and clinical problem solving. Teaching strategies and materials included pre-video self-study, scenario-based learning, practice clinical skills, and team learning using childbirth pelvic models, low-fidelity simulators, and standardized patients.

#### Development phase

Based on the analysis of learning needs and job tasks, four maternal nursing competencies were developed: Assessment of and intervention in (1) postpartum uterine involution and vaginal discharge (uterine involution), (2) amniotic membrane rupture (rupture of membrane), (3) delivery process through vaginal examination (delivery process); and (4) nursing care for patients undergoing induced labor (induced labor). The core skills according to maternal nursing competencies were assessment of and intervention in postpartum uterine involution and vaginal discharge; assessment of and intervention in amniotic membrane rupture; assessment of and intervention in delivery process through vaginal examination; oxytocin drug injection and intervention, and electronic fetal monitoring and intervention. A protocol was developed for each core skill through the analysis of learning needs and job tasks, and the developed protocol was validated by three professors of maternal health nursing.

#### Implementation and evaluation phase

Plans were made for identifying needs that would occur in implementing the 2-day program (Table 1) and evaluation parameters were identified for measuring maternal nursing performance, problem solving, emotional intelligence, and self-directed learning ability.

### Testing the efficacy of the maternal nursing competency reinforcement program

#### Study design

This study used an explanatory sequential mixed method design [14] employing a non-blinded, randomized controlled trial (RCT), followed by open-ended interviews with a subset of program participants. The explanatory sequential mixed method design was employed to sequentially collect and analyze quantitative and qualitative data, in order to understand the effect of the program more abundantly than when only quantitative research design is used [14].

### 1. RCT phase

This study ascribed to the CONSORT (Consolidated Standards of Reporting Trials) guidelines [15].

#### Participants and recruitment

The participants of this study were third year nursing students who had completed their first maternal nursing lecture class and clinical practicum in the first semester, and were taking their second maternal nursing lecture class in the second semester, and had not yet started their maternal nursing clinical practicum. Eligible students from Daegu Catholic University in Daegu, Korea, received an online explanation of the study and one more invitation by a research assistant, following classes. It was explained that refusal to participate would not adversely affect grades, and those who voluntarily expressed their intention to participate were selected as participants.

#### Sample size

**Figure 1.**
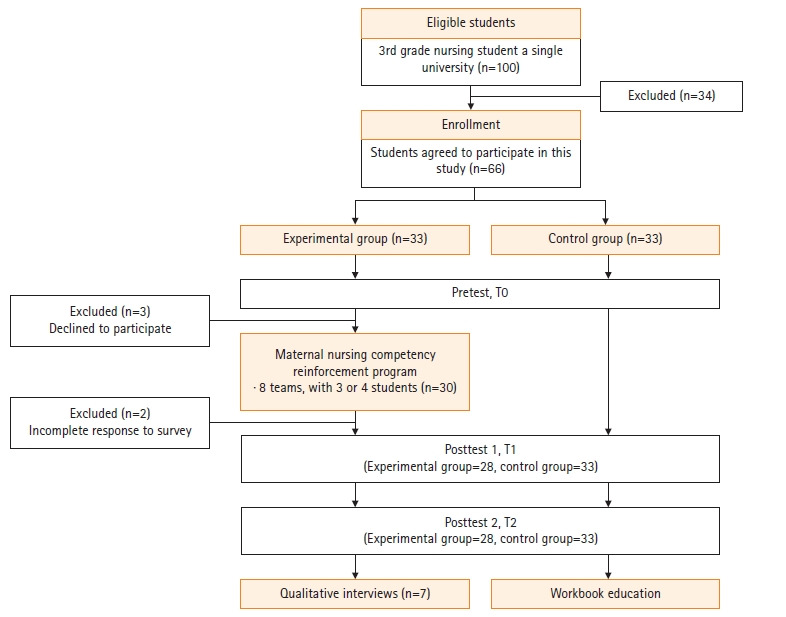
Flow diagram of participants.

Sample size was calculated using the G*Power 3.1.9.2 program, with an effect size of .42, based on Song and Son’s study [16]. For the repeated measures analysis of variance (ANOVA), estimation by an effect size .42, power of .95, and significance level of .05, for two groups across three measurements, and the correlation coefficient between repeated measurements set at .5, at least 52 participants were necessary. In consideration of dropout (25%) based on similar studies [17], we aimed to recruit 66 students. The experimental group and control group were assigned using random numbers, with 33 students in each group. After excluding students who did not participate in the program (n=3) and had incomplete data at the posttest (n=2), data from 61 students (28 experimental, 33 control) were used for final analysis (Fig. 1).

#### Measurement

All structured instruments were used with permission from the developers.

Maternal nursing performance: After reviewing the literature on core maternal nursing skill protocols and general simulation competency evaluation tools for students, the authors developed a maternal nursing performance tool covering four maternal nursing competencies: uterine involution (19 items), rupture of membrane (20 items), delivery process (13 items), and induced labor (15 items). Participants self-evaluate the degree to which they can perform each task on a 5-point Likert (1, not at all to 5, extremely), with higher mean scores indicating greater maternal nursing performance capability. In this study, the Cronbach’s α value were .95 (uterine involution), .94 (rupture of membrane), .93 (delivery process), and .95 (induced labor).

Problem-solving ability: Problem-solving ability was measured using the Adult Problem-solving Process tool that asks about actions one can take when trying to solve problems related to pregnancy and childbirth [18]. This tool is composed of a total of 30 questions, and five subcategories: clarification of the problem (six items), seeking a solution (six items), making a decision (six items), applying the solution (six questions), and evaluation and reflection (six items). Each item is rated on a 5-point Likert (1, not at all to 5, extremely), with higher mean scores indicating greater problem-solving ability. Cronbach’s α was .93 at development [18] and .97 in this study.

Emotional intelligence: We used The Wong and Law’s Emotional Intelligence Scale (WLEIS) [19] that was translated into Korean [20]. The tool has four items each in the following four subcategories: appraisal and expression of emotion in the self, appraisal and recognition of emotion in others, regulation of emotion in the self, and use of emotion to facilitate performance. The mean score rated on a 7-point Likert scale (1, not at all to 7, extremely) is used, with higher scores indicating greater emotional intelligence. At the time of tool development, Cronbach’s α was .87 [19], and .91 in this study.

Self-directed learning ability: The 45-item Self-directed Learning Ability measurement for college students developed by the Korea Educational Development Institute [21] was used in this study. There are three competency areas: learning planning ability (20 items; diagnosing learning needs, setting learning goals, and discovering learning resources), learning execution ability (15 items; basic self-management ability, selection of learning strategies, and continuity of learning execution); and learning evaluation ability (10 items; attribution of effort and self-reflection on the outcome). Using a 5-point Likert (1, very rarely to 5, very often), higher mean scores indicating higher self-directed learning skills. Cronbach’s α was .93 at development [21], and .92 in this study.

#### Procedures

The study was conducted from October 10 to November 16, 2018. Measurements were done before the intervention (T0), immediately afterward (T1), and three weeks later (T2).

Pretest (T0): One week before the program began; pretests were done through an online questionnaire. This included maternal nursing performance, problem-solving ability, emotional intelligence, self-directed learning ability, and demographic characteristics.

Experiment: The experimental group consisted of a total of eight teams, with three or four students on each team. Two maternal nursing professors and two delivery room nurses were assigned as professional instructors for each of the four maternal nursing competencies. The experimental group took part in the maternal nursing competency reinforcement program over the course of 2 days (580 minutes). In the program, a low-fidelity simulator was used to advance nursing skills through genital observation and assessment, and the students practiced interpersonal interaction using standardized patients and student patients who were not research participants. Each team learned two maternal nursing competencies per day. Three days before the program began, an overall orientation was given to introduce the purpose and progress of the program (30 minutes), and individual assignments were given, which included watching videos on core skills (approximately 60 minutes). The experimental group was then asked to study four maternal nursing competencies in a team learning setting over 2 days (480 minutes). Team learning consisted of understanding the scenario understanding (10 minutes), technical demonstration and technical training (60 minutes), and debriefing (50 minutes) (Table 1). A workbook was used during the program, and each team did their activities in a separate training room. The debriefing was conducted by the instructors who led each maternal nursing competency. The control group was provided usual care, general orientation on clinical practicum. After T2 measurement, the control group was provided with the workbook and information on the four maternal nursing competencies. About $3 was offered as an incentive to all students who participated in the study, after completion of T1 and T2.

Posttest (T1 & T2): The first posttest (T1) was conducted immediately upon completion of the intervention through a self-report questionnaire given to both groups at different locations. The second posttest (T2) was conducted three weeks later, with both groups gathered in different classrooms.

#### Data analysis

Analyses were done using IBM SPSS ver. 25.0 (IBM Corp., Armonk, NY, USA). To assess the degree of homogeneity of the experimental and control groups, the chi-square test and Fisher exact test were used for general characteristics, and an independent t-test was used for the outcome variables. Normality was confirmed by the Shapiro-Wilk test both before and after the program. Based on the homogeneity test, variables with significant differences between the two groups (age, and pretest results of delivery process) were treated as covariates, and the main effects were evaluated using repeated measures ANOVA. Mauchly’s sphericity test was used to verify the homogeneity of variance, and if the sphericity assumption was not established, the Greenhouse-Geisser value was taken.

### 2. Qualitative phase

#### Participants

A subsection of the experimental group participants were invited to individual interviews. Selection was based on pre-post differences in maternal nursing performance scores: those who showed a small difference (n=3), those with a large difference (n=3), and one participant with a moderate difference in scores. After analyzing the interview data of seven participants, no further interviews were conducted because no new content was derived. The mean age of participants (six females, one male) was 22.00 years (standard deviation [SD], ±2.01), and the mean academic achievement scores for the previous semester was 80.69 (SD, ±6.86) in maternal nursing lecture class and 90.20 (SD, ±8.0) in maternal nursing clinical practicum.

#### Procedures

Individual interviews were done by one researcher, with a subset of participants in the experimental group, one week after the second posttest via purposive sampling. The interviews ranged from 25-40 minutes and the main interview questions were: “Which maternal nursing competency do you think has improved the most, and why do you think that?”, “What is the least improved maternal nursing competency? And why do you think that?”, and “What kind of nursing problem do you think could be solved if you could care directly for the patient?”.

#### Data analysis

The interview data were analyzed using content analysis method [22]. The sensitivity was increased by the researcher comparing the content with the manuscript and repeatedly listening to the recorded file and ensuring the accuracy of the interview content by referring to the field notes. Each main category, and code were defined. The researchers reviewed the derived meanings several times and reconfirmed the original data. As a final step, the final result was reviewed by two professors experienced in qualitative research and four participants and the final agreement and revision were made through discussion among the research team.

### 3. Integration of quantitative and qualitative findings

Integration in this study involved connecting the results from the quantitative phase to help plan the follow-up qualitative data collection phase. Through this connection, questions needed for further probing were identified, which participants could help best explain the quantitative results.

## Results

### General characteristics and test for homogeneity of the participants

The majority of participants were female (92.9% of experimental group, 93.9% control group) and there was no significant difference between the two groups. The only statistically significant difference (*p*=.001) between the experimental and control groups was for mean age (22.93±2.28 years and 21.21±1.34 years, respectively). The two groups were comparable in academic achievement levels in lecture classes (81.64±7.02 in experimental, 79.85±6.33 in control group) and in clinical practicum (89.46±8.89 in experimental, 90.21±7.48 in control group) (Table 2).

Among maternal nursing performance, uterine involution (*p*=.133), rupture of membrane (*p*=.063), and induced labor (*p*=.057) were not significantly different between the two groups at baseline. However, the score of delivery process was slightly higher in the control (3.75±0.55) than in the experimental (3.37±0.83) at T0 (*p*=.042). Problem-solving ability (*p*=.931), emotional intelligence (*p*=.614), and self-directed learning ability (*p*=.077) were not significantly different between the two groups at T0.

### Effects of the maternal nursing competency reinforcement program on main variables

The program’s effect on maternal nursing performance is presented in Table 3. Uterine involution (F=24.04, *p*<.001), rupture of membrane (F=36.39, *p*<.001), delivery process (F=32.42, *p*<.001), and induced labor (F=48.03, *p*<.001) significantly differed by group×time interaction. Problem-solving ability (F=9.73, *p*<.001) and emotional intelligence score (F=4.32, *p*=.016) significantly differed by group×time interaction. But, self-directed learning ability significantly differed only by group (F=13.09, *p*=.001) (Table 3).

### Qualitative phase findings

Three main categories were identified regarding how this program helped maternal nursing competency, problem-solving ability, emotional intelligence, and self-directed learning ability.

#### Main cateory 1: Learning with a colleague by simulation promotes self-reflection and learning

This was derived from four categories (Table 4). Participants thought that self-driven video-based learning induced more interest in face-to-face learning, and that they were able to concentrate on the material in the videos and learn the content faster. Participants observed and sought out self-reflection and learning strategies while observing the activities of their peers during the program and in the debriefing sessions. Participants said that they were able to review the content after participating in the program, and that it helped them to combine knowledge and practice.

*“…While doing this program, I have been self-directed and re-discovered learning content... and that. Well… Ah! That’s the effacement of the cervix and the opening of a few centimeters. I think it was an opportunity to study in a self-directed manner.” (Participant A)*

However, some participants said that they felt motivated to learn after participating in the program, but they could not translate that motivation into practice.

#### Main category 2: Improvement in maternal nursing knowledge and performance by learning various countermeasures

This main category was derived from eight categories (Table 4). Participants expressed that they were able to improve their nursing abilities by observing various clinical case studies and various coping methods of their peers and that they became familiar with nursing practice and gained confidence through repeated observation. They also thought that the program gave them the opportunity to connect the knowledge and practice of maternal nursing, to identify and to correct uncertain knowledge, and learn how to understand and cope with patients while practicing direct nursing.

*“I think it’s good to do that. Ah! This way, even if I actually go to the hospital (for clinical practicum), I can do it. I can see it!” (Participant C).*

However, some participants recognized that empirical learning in nursing practice was insufficient, as they were not skilled enough to make good clinical decisions despite coming to understand the patient’s situation.

#### Main category 3: Learning of emotionally supportive care, but being insufficient

This was derived from two categories (Table 4). Participants better understood and sympathized with their patients after participating in the program, but they were embarrassed because they could not fully control their own emotions. They still felt that their ability to provide emotionally supportive care to their patients was inadequate.

*“Actually, I’m not good at recognizing other people’s feelings. Now, I understand that the patients are naturally anxious about delivery or her own condition. When the patient rejected it at first, in fact, it was embarrassing and put temporal pressure on what to do at that time. I don’t know the best thing to do in that situation…” (Participant D).*

Some participants said that they felt satisfied when they expressed empathy to the patients and learning to provide emotionally supportive care.

### Integration of findings

Upon integration of findings from both phases, the maternal nursing competency reinforcement program was found to be effective for maternal nursing performance, problem-solving ability, and emotional intelligence. It was also found to be conducive for self-reflection and learning, and knowledge and performance of maternal nursing skills. Learning emotionally supportive care was also noted as a positive experience, although somewhat insufficient.

## Discussion

### Key results

Although the training program lasted for a short period, it had the effect of increasing maternal nursing performance, problem-solving ability, and emotional intelligence over time, which are all quite significant results. In addition, the program was composed of maternal nursing competencies that nursing students must acquire in practical learning in the second semester of the third year; it thus has a strength in that it is tailored to the needs and levels of the students.

### Interpretation

Firstly, the experimental group showed better maternal nursing performance over time than the control group. Interviewees in this study expressed confidence that their nursing abilities had improved because they repeatedly observed their peers’ performance in various cases, and practiced maternal nursing on their own through simulation training. Similarly, in previous studies, simulation-based practice was more effective at improving nursing skills than other learning strategies [23]. The interview data also supported positive experiences of maternal nursing knowledge and perception of improved skills.

The maternal nursing competency reinforcement program also improved the problem-solving ability of nursing students. The interview participants said that although they did not directly experience nursing-related problems in lecture classes or clinical practice, they were able to learn various nursing response strategies and application skills through the case study, peer observation study, and repetitive learning by participating in the program. In previous studies, simulations that applied problem-based learning for maternal nursing were effective at improving maternal nursing performance [24]. In a meta-analysis study of simulation-based learning using standardized patients, the simulation helped students improve their knowledge, communication skills, self-efficacy, and clinical performance [25]. Therefore, it is thought that repetitive direct and indirect behavioral performance learning through simulation-based learning and peer observation learning influence the students’ ability to identify problems, develop solutions, and implement those solutions in the realm of maternal nursing.

Second, improvements were also found for emotional intelligence pre and posttest and interviews showed that students came to understand the mental and physical situation faced by pregnant women. This is in line with ‘appraisal and recognition of emotion in others,’ which is an attribute of emotional intelligence. Students also learned how to deal with their patients’ emotional expression more wisely by observing their colleagues, which reflects ‘use of emotion to facilitate performance,’ another attribute of emotional intelligence. There is a worldwide need for the development of emotional intelligence in nursing education for nurses and midwives [26]. Thus, education that promotes emotional intelligence should be provided to increase the quality of professional nursing and compassionate care [26]. However, until now, there have been no experimental studies aimed at promoting the emotional intelligence of nursing students in maternity-related simulations. Therefore, this study is meaningful in that it attempted to evaluate the effects on emotional intelligence. Emotional intelligence can be developed by performing self-assessment, reflection activities, modeling of emotional intelligence behaviors, and development of empathy, using experiential learning strategies such as simulation training and role play and peer mentoring strategies [27]. It has been pointed out that emotional intelligence education should be integrated into the nursing curriculum and become an ongoing process rather than a short-term, temporary learning [25]. As such, because the scenario-based simulation education strategy was used in this study, emotional intelligence framed in the virtual patient scenarios may have been a safe and natural way for students to understand the patient’s emotions more deeply and train their emotional intelligence. Emotional intelligence education improves the efficiency of nursing care services and professional competence [28]. However, as qualitative findings found that learning emotionally supportive care was somewhat insufficient, more programs that include emotional intelligence as a learning strategy should be developed in the future.

Third, the difference in self-directed learning ability between the experimental and control groups was significant, but the difference in self-directed learning ability over time was not. Self-directed learners should use a structured approach and develop facilitator-guided self-regulated learning skills [29]. Blended coaching, which has been used in self-directed learning programs in clinical practice for two weeks, provided direct guidance both online and offline, feedback on discussions, encouragement for the writing of reflection reports and assignments, and encouragement to review and act on daily learning goals and contents [30]. In this study, the program operation period was short (2 days) and there were few strategic self-directed learning activities. The interviewees in this study recognized that the learning process was promoted by reflecting on their own learning, establishing a learning strategy, and linking knowledge with practice while learning with their peers. However, some participants said that they felt motivated to learn but could not translate that motivation into practice. Therefore, adding coaching to promote self-reflection, assignment of tasks, and facilitate encouragement may be helpful for future educational programs and research.

Fourth, this study is significant in that it presents evidence that can be used in nursing education. In addition, this practical program was developed by investigating maternity nursing-related learning needs of nursing students, analyzing job tasks with experts, and selecting the most important topics. As a tailored maternal nursing competency reinforcement program for third-year students in the second semester, its effectiveness was verified by quantitative study and further strengthened by the narratives from results.

### Limitations

As all participants were students from a single university, the results may be limited in generalizability to other nursing programs. Also, although interviews were done with full assurance of anonymity and no linkage to academic performance, students may not have fully disclosed their honest opinion about any negative aspects of the program.

In conclusion, this 2-day maternal nursing competency reinforcement program was shown to improve maternal nursing performance, problem-solving ability, and emotional intelligence in nursing students learning high-risk maternal nursing care. Interview findings also found it to be conducive for self-reflection and learning, performance of maternal nursing skills, and learning emotionally supportive care. Therefore, this program can be used to strengthen maternal nursing competency. Adding a coaching strategy may strengthen self-directed learning skills and extending the number of program hours may ensure sufficient training.

## Figures and Tables

**Table 1.** Content and organization of the maternal nursing competency reinforcement program

Session	Topic	Content	Teaching strategy	Time (min)
1	Introduction of program	• Greetings, introduction	• Building rapport	30
		• Introducing the program’s goals and operating methods	• Motivating for learning	
			• Guiding to learning management	
2	Preview	Homework	• Facilitating self-directed learning	60
		• Watching the video about core major nursing skill		
3	Uterine involution scenario	1) Understanding the scenario	• Elicit participants to know how to assess maternal uterine involution and postpartum lochia.	10
		• Understanding the symptoms of uterine involution and postpartum lochia, assessing the condition	• Promote participants to understand and empathize with the maternal physical and psychological state.	
		• Empathize with the maternal psychological state when insufficiency of uterine involution	• Encourage participants themselves to derive nursing problems that may arise with uterine involution and postpartum lochia.	
		• Thinking about problem-focused solutions	• Elicit participants to learn the rationale for uterine involution and postpartum lochia.	
		2) Practice clinical skills	• Demonstrate nursing performance that meets the patient’s nursing needs	60
		• Observing the demonstration	• Observe and feedback 1:1 training	
		• 1:1 skill training	• Promote positive reinforcement	
		3) Debriefing	• Elicit reflection and internalization.	50
	Rupture of Membrane scenario	1) Understanding the scenario	• Elicit participants to know how to assess amniotic membrane rupture	10
		• Assessing amniotic membrane rupture	• Promote participants to understand and empathize with the maternal physical and psychological state.	
		• Empathize with the maternal psychological state in case of amniotic membrane rupture or premature amniotic membrane rupture	• Encourage participants themselves to derive nursing problems that may arise with amniotic membrane rupture.	
		• Thinking about problem-focused solutions	• Elicit participants to learn the rationale for amniotic membrane rupture, nitrazine test, and AmniSure^®^ test	
		2) Practice clinical skills	• Demonstrate nursing performance that meets the patient’s nursing needs	60
		• Observing the demonstration	• Observe and feedback 1:1 training	
		• 1:1 skill training	• Promote positive reinforcement	
		3) Debriefing	• Elicit reflection and internalization.	50
4	Delivery process scenario	1) Understanding the scenario	• Elicit participants to know how to delivery progress	10
		• Assessing delivery progress	• Promote participants to understand and empathize with the maternal physical and psychological state.	
		• Empathize with the maternal psychological state during the delivery process and vaginal examination	• Encourage participants themselves to derive nursing problems that may arise with delivery progress.	
		• Thinking about problem-focused solutions	• Elicit participants to learn the rationale for delivery process and vaginal examination.	
		2) Practice clinical skills	• Demonstrate nursing performance that meets the patient’s nursing needs	60
		• Observing the demonstration	• Observe and feedback 1:1 training	
		• 1:1 skill training	• Promote positive reinforcement	
		3) Debriefing	• Elicit reflection and internalization.	50
	Induced labor scenario	1) Understanding the scenario	• Elicit participants to know how to induced labor	10
		• Assessing the need for induced labor	• Promote participants to understand and empathize with the maternal physical and psychological state.	
		• Empathize with the maternal psychological state during the induced labor	• Encourage participants themselves to derive nursing problems that may arise with induced labor.	
		• Thinking about problem-focused solutions	• Elicit participants to learn the rationale for induced labor.	
		2) Practice clinical skills	• Demonstrate nursing performance that meets the patient’s nursing needs	60
		• Observing the demonstration	• Observe and feedback 1:1 training	
		• 1:1 skill training	• Promote positive reinforcement	
		3) Debriefing	• Elicit reflection and internalization.	50
5	Closing	• Presentation of impressions of participation	• Re-promote positive reinforcement	10
		• Distribution of certificate finish		
		• Greetings		
• A total of eight teams, with three or four students on each team (Each team learns two maternal nursing competencies per day)				580
• A 2-day course (a total of four maternity nursing competencies)				

**Table 2.** General characteristics of participants and test of homogeneity (N=61)

Variable/categories	Exp. (n=28)	Cont. (n=33)	χ^2^ or t	*p*
	n (%) or mean±SD	n (%) or mean±SD		
Sex				
Male	2 (7.1)	2 (6.1)	0.03^†^	>0.99
Female	26 (92.9)	31 (93.9)		
Academic achievement level				
Lecture classes	81.64±7.02	79.85±6.33	1.04	.305
Clinical practice classes	89.46±8.89	90.21±7.48	–0.36	.722
Age (years)	22.93±2.28	21.21±1.34	3.65	.001
Problem-solving ability	3.25±0.54	3.24±0.62	0.09	.931
Emotional intelligence	5.08±0.68	4.98±0.80	0.51	.614
Self-directed learning ability	3.44±0.43	3.25±0.39	1.80	.077
Maternal nursing performance				
Uterine involution	3.15±0.62	3.39±0.63	–1.52	.133
Rupture of membrane	3.20±0.75	3.52±0.56	–1.90	.063
Delivery process	3.37±0.83	3.75±0.55	–2.10	.042
Induced labor	3.09±0.80	3.46±0.66	–1.94	.057

Exp.: Experimental group, Cont.: control group.

† Fisher exact test.

**Table 3.** Comparison of outcome variables between the groups (N=61)

Variable	Possible score range	Time	Exp. (n=28), mean±SD	Cont. (n=33), mean±SD	Between groups		Source	F^†^	*p*
					F^a^	*p*			
Problem-solving ability	1–5	T_0_	3.25±0.54	3.24±0.62	0.09	.931	Group	15.36	<.001
		T_1_	3.87±0.41	3.40±0.63	3.49	.001	Time	5.07	.008
		T_2_	4.11±0.49	3.43±0.42	5.79	<.001	G×T	9.73	<.001
		T_1_–T_0_	0.62±0.73	0.16±0.43	8.66	.005			
		T_2_–T_0_	0.86±0.69	0.19±0.50	16.14	<.001			
Emotional intelligence	1–7	T_0_	5.08±0.68	4.98±0.80	0.51	.614	Group	6.22	.016
		T_1_	5.47±0.68	5.06±0.98	1.82	.073	Time	6.31	.015
		T_2_	5.69±0.87	5.06±0.86	2.82	.007	G×T	4.32	.016
		T_1_–T_0_	0.39±0.53	0.08±0.57	2.27	.138			
		T_2_–T_0_	0.61±0.66	0.08±0.68	6.91	.011			
Self-directed learning ability	1–5	T_0_	3.44±0.43	3.25±0.39	1.80	.077	Group	13.09	.001
		T_1_	3.56±0.39	3.40±0.41	1.59	.117	Time	1.64	.199
		T_2_	3.75±0.53	3.34±0.33	3.67	.001	G×T	2.51	.092
		T_1_–T_0_	0.12±0.34	0.15±0.33	0.19	.664			
		T_2_–T_0_	0.31±0.48	0.09±0.28	2.20	.143			
Maternal nursing performance									
Uterine involution	1–5	T_0_	3.15±0.62	3.39±0.63	–1.52	.133	Group	65.81	<.001
		T_1_	4.50±0.34	3.52±0.64	7.67	<.001	Time	3.61	.030
		T_2_	4.42±0.44	3.48±0.63	6.61	<.001	G×T	24.04	<.001
		T_1_–T_0_	1.35±0.71	0.12±0.68	36.55	<.001			
		T_2_–T_0_	1.27±0.64	0.09±0.73	31.54	<.001			
Rupture of membrane	1–5	T_0_	3.20±0.75	3.52±0.56	–1.90	.063	Group	58.44	<.001
		T_1_	4.56±0.48	3.73±0.56	6.10	<.001	Time	12.71	<.001
		T_2_	4.55±0.46	3.67±0.57	6.35	<.001	G×T	36.39	<.001
		T_1_–T_0_	1.27±0.64	0.21±0.42	53.18	<.001			
		T_2_–T_0_	1.35±0.81	0.17±0.50	44.01	<.001			
Delivery process	1–5	T_0_	3.37±0.83	3.75±0.55	–2.10	.042	Group	38.46	<.001
		T_1_	4.56±0.41	3.80±0.72	5.12	<.001	Time	3.45	.035
		T_2_	4.56±0.46	3.82±0.55	5.68	<.001	G×T	32.42	<.001
		T_1_–T_0_	1.18±0.90	0.05±0.47	44.65	<.001			
		T_2_–T_0_	1.19±0.80	0.06±0.44	47.27	<.001			
Induced labor	1–5	T_0_	3.09±0.80	3.46±0.66	–1.94	.057	Group	9.40	.003
		T_1_	4.42±0.41	3.57±0.67	6.01	<.001	Time	14.98	<.001
		T_2_	4.47±0.42	3.56±0.57	7.21	<.001	G×T	48.03	<.001
		T_1_–T_0_	1.33±0.79	0.12±0.53	47.95	<.001			
		T_2_–T_0_	1.38±0.77	0.10±0.44	61.77	<.001			

Exp.: Experimental group, Cont.: control group, T_0_: pretest, T_1_: posttest 1, T_2_: posttest 2, G×T: group×time interaction.

† Age and pretest results of delivery process were adjusted.

**Table 4.** Qualitative findings on having experienced the maternal nursing competency reinforcement program

Main category	Categories
Learning with a colleague by simulation promotes self-reflection and learning	Self-driven video-based learning conducted in advance was a catalyst for learning
	Self-reflection and learning strategies by observing peer activities and participating in debriefings
	Reviewing the learning contents and incorporating the knowledge with the nursing practices
	Being motivated to learn, but having challenges translating the motivation into real practice
Improvement in maternal nursing knowledge and performance by learning various countermeasures	Learning a variety of countermeasures through peer observation
	Improving nursing practice through various clinical case studies
	Improving nursing practice through continuous learning
	Increasing confidence in the ability to solve problems related to maternal nursing
	Linking maternal nursing knowledge to practice
	Correcting inaccurate nursing knowledge
	Understanding the patient and managing through direct nursing
	Insufficient practical learning
Learning of emotionally supportive care, but somewhat insufficient	Understanding the patient’s emotions but being unable to respond
	Feeling empathy learning confidence in emotionally supportive care

## Supplementary materials

Further details on supplementary materials are presented online (available at https://doi.org/10.4069/kjwhn.2021.09.13).
