# Supplementary figures and images for: The effects of a maternal nursing competency reinforcement program on nursing students’ problem-solving ability, emotional intelligence, self-directed learning ability, and maternal nursing performance in Korea: a randomized controlled trial
Source: Korean J Women Health Nurs. 2021 Sep 30;27(3):230–42. doi: 10.4069/kjwhn.2021.09.13 (PMC9328593; doi:10.4069/kjwhn.2021.09.13)

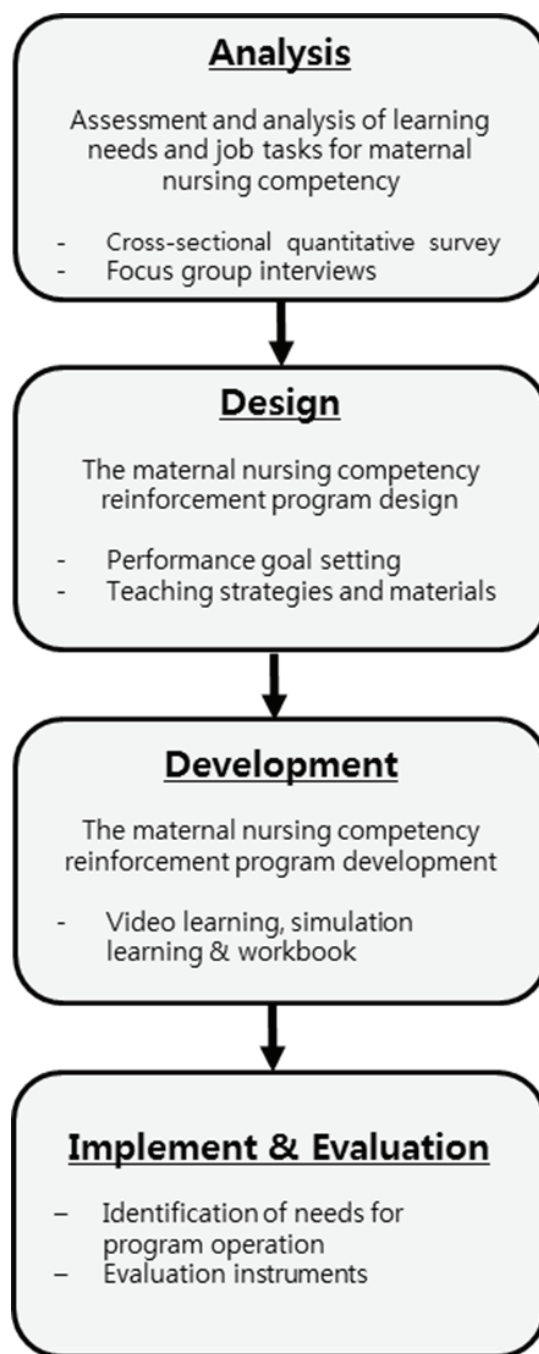

Supplementary Fig. 1. Research procedures.

Supplement: Supplementary file 1 [file kjwhn-2021-09-13-suppl.pdf]
